# Relationship between *Chlamydia-Ureaplasma-Mycoplasma* Genital Detection with Semen Concentration and Motility among Greek Men

**DOI:** 10.22074/ijfs.2017.4690

**Published:** 2017-02-16

**Authors:** Ageliki Gerovassili, Ourania Marcandona, Byron Asimakopoulos, Vasilis Karavasilis, Maria Panopoulou, Alexandros Ikonomidis

**Affiliations:** 1 Biogonidiaki, Center of Infertility Investigation and Genetic Research, Volos, Greece; 2 Democritus University of Thrace, Laboratory of Physiology, Alexandroupolis, Greece; 3 IASO General Hospital, Department of Microbiology, Larissa, Greece; 4 Democritus University of Thrace, Department of Microbiology, Alexandroupolis, Greece

**Keywords:** Polymerase Chain Reaction, Oligozoospermia, Asthenozoospermia, Azoospermia, Infertility

## Abstract

One hundred and seventy two men at the State of Thessaly, Greece, inquiring semen analysis were enrolled in the study in order to investigate the incidence of *Chlamydia, Ureaplasma and Mycoplasma* (C-U-M) genera in respect to total sperm number (TSN), progressive motility (grades a and b) and total motility (grades a, b and c). Putative relation of C-U-M acquirement with sexual behavior was also investigated. Incidence of C-U-M among non-oligozoospermic and oligozoospermic men was similar. Νο correlation of C-U-M carriage to either oligozoospermia or asthenozoospermia was found. The tested semen parameters were negatively correlated to the age of sexual intercourse initiation and positively correlated to the number of sex partners. Early age of sexual intercourse initiation or high number of sexual partners was not statistical significantly correlated to C-U-M acquirement. Overall, TSN and motility (either progressive or total) were not influenced by the presence of C-U-M genera in a sample of Greek population undergoing semen evaluation. To distinguish the role of C-U-M in male infertility and clarify the so far controversial scarce literature, large control case studies are needed using nucleic acid amplification techniques to detect these pathogens.

Up to date, epidemiological studies have shown that infertility affects approximately 10% of the couples, whereas in 50% of this incident, a male infertility associated factor is found (1). A thorough sexual/medical history as well as hormonal/ physical examination may reveal the cause of male infertility in 70% of infertile men. Of the remaining 30%, the vast majority of infertile males may carry genetic abnormalities such as Robertsonian translocations, Y chromosome micro/macrodeletions, aneuploidy as in Klinefelter syndrome or mutations in the cystic fibrosis transmembrane regulator gene (2).

Among the factors that might influence male fertility, infections of the lower genital tract have been the least investigated field. Effects of urogenital tract pathogens on sperm concentration and motility have still remained unclear and further studies are needed to evaluate their influence on male fertility. Particularly, the *Chlamydia, Ureaplasma and Mycoplasma* (C-U-M) genera are considered sexually transmitted pathogens (although U. and M. are also suggested to constitute normal flora) that might cause chronic urogenital tract infections. Their asymptomatic carriage as well as the low specificity and sensitivity of conventional microbiological methods for C-U-M detection, compared to nucleic acid amplification techniques such as polymerase chain reaction (PCR) (3), might explain the still poorly evaluation of the roles of these pathogens in male fertility. We have recently shown the incidence of C-U-M in Greek population and the prevalence of U. spp. as well as the potential pathogenicity of *U. urealyticum* (4). The aim of the present study was to investigate the presence of C-U-M among Greek men in respect to TSN, sperm motility and sexual behavior in order to investigate the putative effect of these pathogens in semen parameters that might influence conception.

From August 2013 until July 2015, a total of 172 men at the State of Thessaly, Greece, participated in the study. All participants enrolled in the study for semen analysis either inquired a microbiological evaluation (mainly due to clinical manifestations of genital tract infection or preventive screening), or were reported as being infertile after at least a 12-month unprotected sexual intercourse and failure to impregnate their wives. Informed consent was obtained from all participants and each individual filled in a questionnaire regarding demographic data, medical record and sexual history. Males with an underlying pathology (e.g. varicocele and hormone deficiency) to which oligoazoo- or asthenospermia could be attributed were excluded. Duplicate samples from individuals, even if surgical or medical treatment was applied, were not included in the study. Eventually, participants were divided into two groups according to TSN and irrespectively to reason of enrollment in order to study the prevalence of C-U-M in respect to sperm concentration and motility.

Semen samples of all participants were collected into sterile nontoxic recipients by masturbation after 3 to 5 days of sexual abstinence. All participants were evaluated according to the guidelines of World Health Organization (WHO) 2010 Semen Analysis Reference Limits (5), and TSN, progressive motility (grades a and b) and total motility (grades a, b and c) were determined. All semen samples were investigated for DNA of C-U-M using Amplisens *C.trachomatis/U./M.genitalium*-MULTIPRIME- FRT and Amplisens M. hominis-FRT diagnostic CE-IVD PCR kits (Amplisens, Slovak Republic). These kits provide *in vitro* nucleic acid amplification qualitative tests for the detection of DNA of C. trachomatis, *U.* spp. (*U. parvum* and *U. urealyticum*), and *M. genitalium* simultaneously, as well as *M. hominis*, in the clinical material using real-time hybridization-fluorescence detection. They are intended for *in vitro* diagnostic use and are Conformite Europeene (CE) marked. C-U-M DNA extraction was performed using DNA-Sorb- AM Nucleic Acid Extraction Kit (Amplisens, Slovak Republic) which includes Internal Control-FL (IC) reagent in order to test both efficacious DNA extraction and putative inhibition of PCR. Analyses were performed at BIOGONIDIAKI Center of Infertility Investigation and Genetic Research, Volos, Greece that serves for the broad region of the State of Thessaly.

Mean and median values as well as SD were calculated and are given as mean ± SD or median. Unpaired t test, Fisher’s exact test, odds ratios (ORs), 95% confidence intervals (CI), as well as simple linear regression analysis (Pearson r) were performed using statistical software Statistical Package for the Social Sciences (SPSS, SPSS Inc., USA). Statistically significant difference was defined as the P<0.05.

Sixty-eight participants of the study had TSN≥39 millions (control group) and 104 were oligo- azoospermic with TSN<39 millions (study group). In the control group, mean age was 36.66 ± 5.96, mean age of sexual initiation was 18.19 ± 3.15 and median number of sexual partners was 6. In the study group, mean age was 37.42 ± 5.50, mean age of sexual initiation was 19.11 ± 3.69 and median number of sexual partners was 6. Mean TSN of the 68 males of the control group was 258.59 ± 197.32 millions. Progressively motile spermatozoa, more than 32%, were present in 46 participants (67.65%) and totally motile spermatozoa, more than 40%, were present in 63 (92.65%). C-U-M DNA was present in 10 participants (14.71%). Particularly, 8 (11.76%) were positive for U. spp., two (2.94%) for *M. hominis* and two (2.94%) were found to cocarry U. spp. and *M. hominis*. Mean TSN of the 104 males of the study group was 13.69 ± 12.67 millions, which was shown to be statistically significant different (P<0.0001) from the TSN of the control group using unpaired t test. Progressively motile spermatozoa, more than 32%, were present in 20 participants (19.23%), and totally motile spermatozoa, more than 40%, were present in 40 (38.46%). C-U-M DNA was present in 10 participants (9.62%). Particularly, one (0.96%) was positive for *C. trachomatis*, seven (6.73%) for U. spp. and two (1.92%) for *M. hominis* (Table 1). Of note, no M. genitalium-positive participant was found in either the control or the study group.

**Table 1:** Prevalence of the detected microorganisms among the participants of the study


Species	Participants with TSN	
	≥39 millions of spermatozoa n (%)	<39 millions of spermatozoan (%)

C. trachomatis	0 (0.00)	1 (0.96)
U. spp.	8 (11.77)	7 (6.73)
M. hominis	2 (2.94)	2 (1.92)
U. spp.	2 (2.94)	0 (0.00)
M. hominis		


TSN; Total sperm number, C. trachomatis; Chlamydia trachomatis, U. spp.; Ureaplasma species, and M. hominis; Mycoplasma hominis.

Fisher’s exact test and calculation of ORs showed no higher probability of C-U-M carriage in men with low TSN (<39 million of spermatozoa) (P=0.17, OR=0.53 with 95% CI=0.21-1.29) or low sperm motility [progressive motility less or equal to 32% (P=0.15, OR=0.50 with 95% CI=0.20-1.23) and total motility less or equal to 40% (P=0.49, OR=0.66 with 95% CI=0.26-1.72), respectively]. There was no relation between C-U-M acquirement with age of sex initiation (cut off value more than and less or equal to 18) (P=1.00, OR=1.11 with 95% CI=0.45-2.74) or number of sex partners (cut off value more than and less or equal to 6) (P=0.82, OR=0.90 with 95% CI=0.37-2.20). Linear regression analysis showed negative correlation between age of sexual intercourse initiation with TSN (Pearson r=-0.918), sperm progressive motility (Pearson r=-0.666) and sperm total motility (Pearson r=-0.686). Also, positive correlations were shown for the number of sex partners with TSN (Pearson r=+0.493), sperm progressive motility (Pearson r=+0.125) and sperm total motility (Pearson r =+0.387).

In our study, nο correlation of C-U-M carriage to either oligo-azoospermia or asthenospermia was found. It should be noted that when the participants of the study were clustered not according to TSN but according to sperm concentration (semen conc.≥or <15 millions/ml, respectively), the statistical analysis showed no variances. Similar negative and positive correlation coefficients in regression analysis and P>0.05 in chi-square tests were obstructed (statistical analysis and statistical data not shown). Our findings are in accordance with Al-Sweih et al. (6) who found no statistically significant correlation of C-U-M carriage to male infertility (although other parameters such as leukocytospermia were shown to be influenced) studying a total of 127 infertile and 188 fertile men in Kuwait with a PCR-based detection protocol. Our results also comply with Gdoura et al. (7) who did not associate C-U-M carriage with either oligozoospermia or asthenozoospermia, although they found a higher prevalence of *C. trachomatis* among male partners of infertile couples. On the contrary, Liu et al. (8) correlated *U. urealyticum* infection to oligozoospermia in 621 Chinese infertile men using culture based microbiological procedures though. Similarly, Lee et al. (9) found that progressive motility and vitality were significantly lower in *U. urealyticum* positive men, while low total motility and total motile sperm count were significantly related to the presence of *M. hominis*. However, they tested 50 infertile couples and 48 fertile couples with Mycofast Evolution 2 commercial kit (International Microbio, France), a culture medium based assay for the detection of *U. urealyticum* and *M. hominis*. The controversial results reported on the current scarce literature are probably related to the diversity of the detection methods used.

Under the scope of a possible correlation of sexual behavior to C-U-M acquirement that might influence semen quality, we found that semen parameters were correlated negatively to the age of sexual intercourse initiation and positively to the number of sex partners. In other words, the earlier a man initiated his sexual life and the more sexual partners he had, the better tested semen parameters he appeared to have. Moreover, the early age of sexual intercourse initiation or the high number of sexual partners was not statistical significantly and correlated to C-U-M acquirement.

In conclusion, TSN and sperm motility seem not to be influenced by the presence of C-U-M genera in a sample of Greek men undergoing semen evaluation. Although prevalence of C-U-M in our sample was low [as was expected due to a lately published large-scale study from Central Greece (4)], C-U-M appeared to be equally spread to both groups of the study which further highlights the doubtful role of C-U-M in the tested semen parameters. Furthermore, early onset of sexual intercourse and high number of sexual partners were not correlated with C-U-M acquirement. To distinguish the role of C-U-M in male infertility and clarify the so far controversial scarce literature, larger case control studies are needed using nucleic acid amplification techniques to detect these pathogens, as recent reviews have suggested (10).
